# Determination of radiotherapeutic target zones for thoracic esophageal squamous cell cancer with lower cervical lymph node metastasis according to CT-images

**DOI:** 10.18632/oncotarget.9094

**Published:** 2016-04-28

**Authors:** Xingde Li, Jin Zhao, Ming Liu, Fushan Zhai, Zhengfei Zhu, Feng Yu, Mingyun Zhang, Lijie Han, Yue Zhao, Haiyan Wang

**Affiliations:** ^1^ Cangzhou Central Hospital, Cangzhou, China; ^2^ Hospital No. 3 of Hebei Medical University, Hebei, China; ^3^ Tumor Hospital of Fudan University, Shanghai, China; ^4^ People's Hospital of Qidong City, Nantong, China

**Keywords:** esophageal cancer, radiotherapy, lower cervical lymph node, CT-images, supraclavicular zone

## Abstract

Esophageal squamous cell carcinoma (ESCC) is a leading cause of cancer-related deaths worldwide. And radical synchronized chemoradiotherapy has become an important treatment measures for this disease. It is necessary to define the therapeutic target zone based on computer tomography(CT)-images for precise radiotherapy. Therefore, we retrospectively analyzed the regularity of lymph node metastasis in lower cervical section of thoracic esophageal cancer based on CT-images and discussed the range of radiotherapy in supraclavicular zone. The lower cervical lymphatic drainage area was divided into cervical tracheoesophageal groove (CTG), medial supraclavicular zone (MSC zone) and lateral supraclavicular zone (LSC zone) based on CT-images. We found that the rate of lymph node metastasis to medial CTG and MSC zone was relatively high. And rate of lymph node metastasis to the above two zones from middle thoracic section was on an increasing trend with the progress of T stage. Patients at stage T3 and T4 with lymph node metastasis in tracheoesophageal groove in middle thoracic section showed a higher rate of lymph node metastasis in MSC zone. These results demonstrated that the CTG and MSC zone should be clinically included in the supraclavicular target zone for radical radiotherapy, and the T-stage and tumor location should be considered simultaneously.

## INTRODUCTION

Esophageal cancer continues to be one of the most common malignancies worldwide [1] as well as the third most common gastrointestinal malignancy. And squamous cell carcinoma (SCC) is still the predominant form of the disease. The main causes of treatment failure for this disease are local uncontrol and distant metastasis. Some researchers have found that the 5-year survival rate of chemoradiation for esophageal cancer is close to surgery [2–3]. Therefore, radical synchronized chemoradiotherapy has become an important treatment measures for esophageal cancer [4]. But there is no consensus about the determination of radiotherapeutic target zones for esophageal cancer, especially it is of great controversy at present whether the selective lymph node irradiation (ENI) is needed for radical radiotherapy [5, 6].

Lymph node status is one of the most important factors that can affect the prognosis of esophageal cancer patients [7]. And it is common for these patients to appear with the supraclavicular lymph node metastasis. Domestic scholars reported that the rate of supraclavicular lymph node metastasis accounted for about 7.1% to 15.4% in patients with esophageal cancer when they receive the first radiation therapy, and the 5-year survival rate for these patients with or without supraclavicular lymph node metastasis was 8.2% and 13.7%, respectively(P<0.05). Radiation therapy is the effective way to treat the patients with supraclavicular lymph node metastasis.

According to the traditional set of the boundaries of 2D radiotherapeutic zone for supraclavicular field, the annular cartilage or the cricothyroid membrane, and the line at 1cm below the lower edge of the subclavian head are respectively the upper and the lower bound [8]. While the junctions between the middle 1/3 and the outer 1/3 of each clavicle are two lateral bounds [9]. In the current era of precise radiotherapy, the target zone for clinical radiotherapy is usually defined by means of CT-images. However, there is no uniform definition and sketch of the supraclavicular zone. Therefore, we retrospectively analyzed the regularity of spontaneous metastasis to lower cervical lymph nodes in 386 thoracic esophageal cancer patients to explore the pattern of supraclavicular lymph node metastasis and find out the high-risk zones of supraclavicular lymph node metastasis. And finally we hope to provide a proposal for reference to define the clinical target zones for supraclavicular radiotherapy of esophageal cancer.

## RESULTS

### Distribution characteristics of lymph node metastasis for different zones in lower neck of thoracic esophageal cancer patients

According to CT-based criteria for diagnosis of lymph node metastasis, the rate of lymph node metastasis in CTG, MSC and LSC zones was 47.2% (182/386), 23.1% (89/386) and 4.1% (16/386) respectively in 386 thoracic esophageal squamous cell carcinoma patients. The rate of lymph node metastasis to cervical tracheoesophageal groove from upper, middle and lower thoracic section was respectively 67.2%, 37.9% and 18.8%, of which the rate from upper thoracic section was significantly higher than that from other thoracic sections (X^2^=58.634, P=0.000). In all cases, the rate of lymph node metastasis from upper, middle and lower thoracic sections to MSC zone was 31.6%, 18.9% and 11.3% respectively (X^2^=14.721, P=0.001). And the rate from upper thoracic section was significantly higher than the other two sections (P=0.001), which was similar to the CTG zone. The rate of lymph node metastasis from upper, middle and lower thoracic sections to LSC zone was 6.3%, 3.0% and 1.3% respectively, and there was no significant difference for this zone (X^2^=4.175, P=0.124). The lymph node metastasis occurred directly from upper thoracic section to LSC zone only in three cases while in the rest cases the lymph node metastasis occurred both in LSC and MSC zones. Table 3 showed the lymph node metastasis to different cervical zones of 386 cases with thoracic esophageal cancer.

**Table 1:** Clinical data of 386 patients of thoracic esophageal cancer

Features	The number of patients	Cases with lymph node metastasis	X^2^	P
Gender				
male	209	72	0.123	0.726
female	177	64		
Age				
≤60	157	53	0.252	0.615
>60	229	83		
Location of cancer				
upper thoracic section	174	80	17.528	0.000
middle thoracic section	132	39		
lower thoracic section	80	17		
T stage of tumor				
T1	20	5	1.935	0.586
T2	91	35		
T3	129	42		
T4	146	54		

**Table 2:** Bound marks for CT image-based zoning of supraclavicular lymph nodes of patient with esophageal cancer

Zones	Upper bound	Lower bound	Interior bound	Outer bound	Front bound	Rear bound
CTG	The upper edge of the annular cartilage	The lower edge of the external jugular vein	Outer wall of trachea and esophagus	The inside of the internal carotid artery	Thyroid trailing edge, the leading edge of the trachea	Paraspinal muscles
MSC	The upper edge of the annular cartilage	Outer edge of junction of internal jugular and subclavian vein, the lower edge of the external jugular vein	The inside of the internal carotid artery	Ligature from the rear edge of sternocleidomastoid muscle to outer edge of anterior scalenus muscle	Dorsal side of sternocleidomastoid muscle	Ventral side of anterior scalenus muscle, dorsal side of internal carotid artery
LSC	Outer bound of supraomohyoid muscle	Lower edge of external jugular vein/transverse cervical artery	Outer bound of upper zone of medial supraclavicular	Ribs or trapezius	Clavicle/Skin	Ventral side of Supraomohyoid/levator scapula/medial scalene

**Table 3:** Analysis of lymph node metastasis to cervical zones from thoracic sections of 386 cases

Metastasis location	Upper thoracic section (174)	Middle thoracic section (132)	Lower thoracic section (80)	Total	*P*
CTG zone	117(67.2%)	50(37.9%)	15(18.8%)	182(47.2%)	0.000
MSC zone	55(31.6%)	25(18.9%)	9(11.3%)	89(23.1%)	0.001
LSC zone	11(6.3%)	4(3.0%)	1(1.3%)	16(4.1%)	0.124

Correlation between the rate of lymph node metastasis in different locations and T stages of the cancer

As the results shown in Table 4, it indicated that the correlation between the rate of lymph node metastasis and the T stages. The rate of lymph node metastasis to CTG and MSC zone from middle thoracic section of esophageal cancer was on an increasing trend with the progress of T stages, though the correlation coefficients were only 0.266 and 0.239 respectively by spearman rank correlation analysis. The T stages of esophageal cancer in upper and lower thoracic sections were not statistically correlated with the rate of lymph node metastasis (P>0.05) to medial CTG and MSC zones. But the rate of lymph node metastasis to MSC zone from upper thoracic section was as high as 31.6%, whereas the rate of metastasis to MSC zone from lower thoracic section was only 11.3% (Table 3).

**Table 4:** Correlation between the rate of lymph node metastasis in lower neck and the T stages of cancer

Locations	T1	T2	T3	T4	Correlation coefficient*	P
Upper thoracic section						
CTG	5/8(62.5%)	40/58(69.0%)	28/49(57.1%)	44/59(74.6%)	0.055	0.474
MSC	2/8(25.0%)	22/58(37.9%)	13/49(26.5%)	18/59(30.5%)	−0.048	0.529
Middle thoracic section						
CTG	2/12(16.7%)	3/23(13.0%)	18/42(42.9%)	27/55(49.1%)	0.266	0.002
MSC	0/12(0)	1/23(4.3%)	9/42(21.4%)	15/55(27.3%)	0.239	0.006
Lower thoracic section						
CTG	-	4/10(40.0%)	5/38(13.2%)	6/32(18.8%)	−0.078	0.493
MSC	-	3/10(30.0%)	4/38(10.5%)	2/32(6.3%)	−0.191	0.091

* Coefficient of correlation means Spearman rank correlation coefficient

### Relationship between the lymph node metastasis in lower CTG and MSC zones at different T stages in middle thoracic section esophageal cancer

As we have concluded that the rate of lymph node metastasis to CTG and MSC zone from middle thoracic section of esophageal cancer was on an increasing trend with the progress of T stages, we further explored the relationship between the lymph node metastasis in lower CTG and MSC zone at different T stages in middle thoracic section. The results were shown in Table 5. It indicated that patients with lymph node metastasis to MSC zone accounted for 40% (20/50) of the patients with lymph node metastasis to CTG zone (P=0.000) in middle thoracic section totally. In addition, the lymph node metastasis to MSC zone occurred to about 38.9 % (7/18) and 44.4% (12/27) respectively in patients who were classified as stage T3 and T4 accompanied with the metastasis to CTG zone in middle thoracic section of esophageal cancer (P=0.025 and 0.007). These results implied that lymph node metastasis to tracheoesophageal groove is one of the risk factors for medial supraclavicular lymph node metastasis. But the same conclusion was not drawed at the stage T1 or T2 (P=0.130).

**Table 5:** Relationship between the lymph node metastasis in lower CTG zone at different T stages of esophageal cancer and the lymph node metastasis in MSC zone (cases) in middle thoracic section

	T1		T2		T3		T4		Total	
	CTG a(−)	CTG (+)	CTG a (−)	CTG (+)	CTG a (−)	CTG (+)	CTG a (−)	CTG (+)	CTG a (−)	CTG (+)
MSC (−)	10	2	20	2	22	11	25	15	76	30
MSC(+)	0	0	0	1	2	7	3	12	5	20
P*	-		0.130		0.025		0.007		0.000	

* *P* value is the possibility calculated from Fisher's exact test.

## DISCUSSION

Currently there have no boundaries of lymphatic drainage area for radiotherapy in supraclavicular lymph node zone of esophageal cancer, which was with reference to the recommended treatment for head and neck cancer [15, 16]. The changes of upper limb position will affect the position of lymph nodes during the localization and radiotherapy [17]. Therefore, we analyzed the spontaneous distribution patterns of lymph node metastasis in lower neck of newly diagnosed esophageal cancer patients for radiation therapy under fixed position, with reference to main markers of muscles, bones and blood vessels.

The characteristics of the lymph node metastasis to lower neck of thoracic esophageal cancer were found as follows in this study. (1) The lymph node metastasis to lateral supraclavicular zone was at a low incidence of 4.1% (16/386) in this study, which was parallel to the medial supraclavicular zone in most cases. However, further study is needed to explore whether metastasis to the two zones is correlated to each other. (2) The target sites for lymph node metastasis of esophageal cancer were dominantly the MSC and CTG zones. As shown in Table 3, the rates of lymph node metastasis to the MSC (23.1 %, 89/386) and CTG (47.2%, 182/386) zones indicated an increasing trend from lateral to medial zone. Udagawa et al. [18] reported that the lymph node metastasis occurred mostly along tracheal/laryngeal nerve chain after three-field lymph node dissection, then around the scalene in front of the internal jugular vein, and in transverse cervical artery lymph node at last. The similar pattern was also observed in our study. Therefore, only the MSC and CTG zones are the target zones in clinical target volume (CTV) if prophylactic irradiation to supraclavicular zone is necessary for thoracic esophageal cancer patients.

Some studies have proven that the maximal tumor diameter of esophageal cancer in CT-images directly reflects the range of tumor infiltration. And the deeper the tumor invades, the more opportunities tumor cells can invade into lymphatic vessels [19–21]. In our study, we have proven that T stage of tumor in upper thoracic section was insignificantly correlated with the rate of lymph node metastasis to medial CTG and MSC zone (P> 0.05). However, the rate of lymph node metastasis to the two zones was both relatively high. Therefore, it is recommended to perform the preventive irradiation in both the CTG and MSC zones for this section regardless of its T stage. The rate of lymph node metastasis to CTG and MSC zones from middle thoracic section was on an increasing trend with the progress of T stage. Furthermore, the rate of lymph node metastasis to MSC zone was relatively high in patients who were classified as stage T3 and T4 accompanied with the metastasis to CTG zone in middle thoracic section. These results implied that lymph node metastasis to tracheoesophageal groove is one of the risk factors of medial supraclavicular lymph node metastasis. Therefore, it is necessary to define the individualized supraclavicular target zone for radical radiotherapy in middle thoracic section of esophageal cancer according to T stage and the presence of lymph node metastasis to CTG zone.

There were several shortcomings in this study however. Firstly, the present study was a single center retrospective study. Secondly, the correlation analysis of differentiation degree of tumor tissue was not performed due to the lack of pathological report of the cellular differentiation of tumor in some patients. Moreover, the diagnosis of lymph node metastasis in this study was based on iconographic data instead of pathological evidence. However, the specificity of diagnosis of lymph node metastasis based on CT-images can reach 95% [23] to 98.47% [5] when the short diameter of the lymph node is more than 10mm or the lymph node is visible in CTG zone. Unlike surgery, we couldn't make a pathological diagnosis for each lymph node for the patients received radiotherapy. CT is thebasisof radiation targetvolume delineation. Therefore, the iconographic diagnosis of lymph node metastasis is credible.

In short, the delineated supraclavicular target zone for radiotherapy of esophageal cancer should be selected according to individual clinical situation in need of prophylactic irradiation. The target zones for prophylactic irradiation should include the bilateral MSC and CTG zones as recommended. It is necessary in the future to retrospectively analyze the distribution pattern and clinical correlation of esophageal cancer recurrence in lymph node draining area, and to conduct prospective clinical study on the definition of individualized target area delineation in supraclavicular zone.

## MATERIALS AND METHODS

### Clinical data

Data of 386 patients with newly diagnosed esophageal cancer and treated in radiotherapy department of Cangzhou Central Hospital from October 2009 to August 2014 was collected. All cases, including 209 males and 177 females, with a median age of 58 years (40-81 years), were definitely diagnosed by pathology of esophageal squamous cell carcinoma. Among them, there were 174 cases with cancer in upper thoracic section, 132 cases in middle thoracic section and 80 cases in lower thoracic section. According to clinical staging criteria (draft) of Chinese non-surgical treatment of esophageal cancer [22], there were 20, 91, 129 and 146 cases respectively at the stage of T1, T2, T3 and T4 in order. The clinical data of these patients is shown in Table 1 in detail.

### Radiotherapy methods

The position of all patients was fixed with thermoplastic omentum after both hands held the contralateral elbow and raised to contact forehead. Then the patients received the enhanced cervical and thoracic CT scan ranging from cricothyroid membrane to costophrenic angle horizontal, with the slice thickness of 5 mm. The CT-images were reconstructed into 3-D images and transmitted into the planning system (USA, Eclipse) after digitization.

### Criteria for defining lymph node metastasis

(1) Short diameter of lymph node in soft-tissue window is longer than 10 mm on enhanced CT-images, or there are more than three lymph nodes in the same diagnostic zone [10];

(2) Nodules formed in tracheoesophageal groove.

Meeting one of the above two points can be diagnosed as lymph node metastasis [11, 12]. At least one physician from radiotherapy department and one from radiology department read CT images individually in the diagnosis of lymph node metastasis. Or, the images should be read and discussed by a physician of higher rank in case of controversial reading until a consensus for diagnosis is concluded and then recorded.

### Lymph node zoning

**Figure 1:**
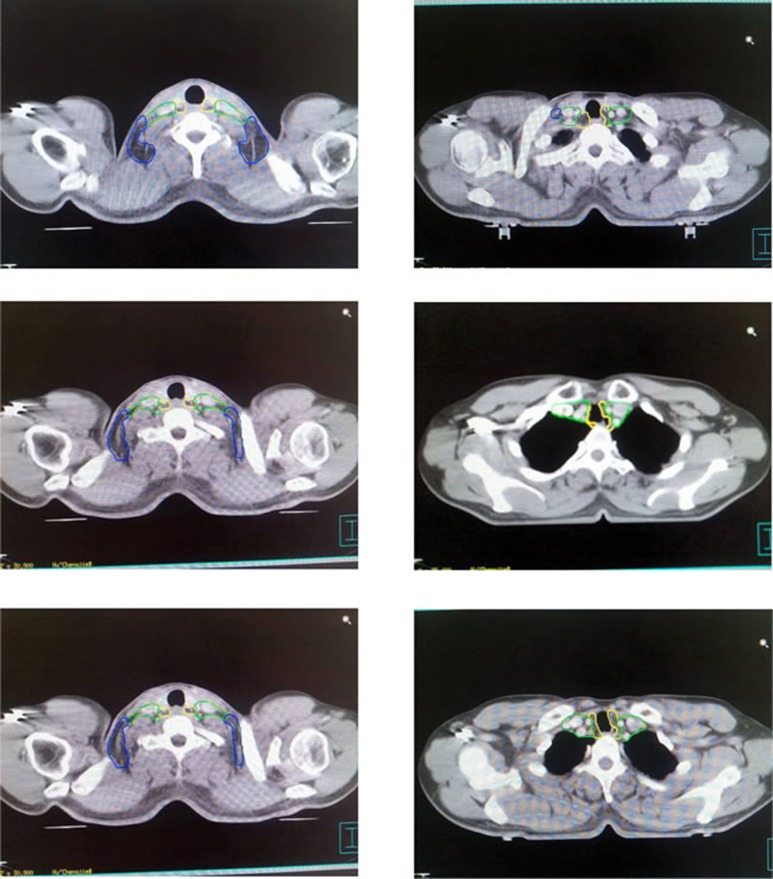
Schematic diagrams of zoning at different bedding planes of CT-imaging in lymphatic drainage area Note: Zones encircled with yellow line are CTG zones, those with green line are MSC zones and those with blue line are LSC zones.

According to the reported method [13, 14], with muscles, bones and blood vessels as the main marker points, the lymph nodes in lower neck of esophageal cancer were zoned as long as they can be delineated based on CT-images in principle. In addition, the zones were divided into three zones, cervical tracheoesophageal groove (CTG zone), medial supraclavicular zone (MSC zone) and lateral supraclavicular zone (LSC zone). Moreover, six bounds for each zone were defined in Table 2. And the schematic diagrams of zoning at different CT bedding planes were indicated in Figure 1.

**Figure 2:**
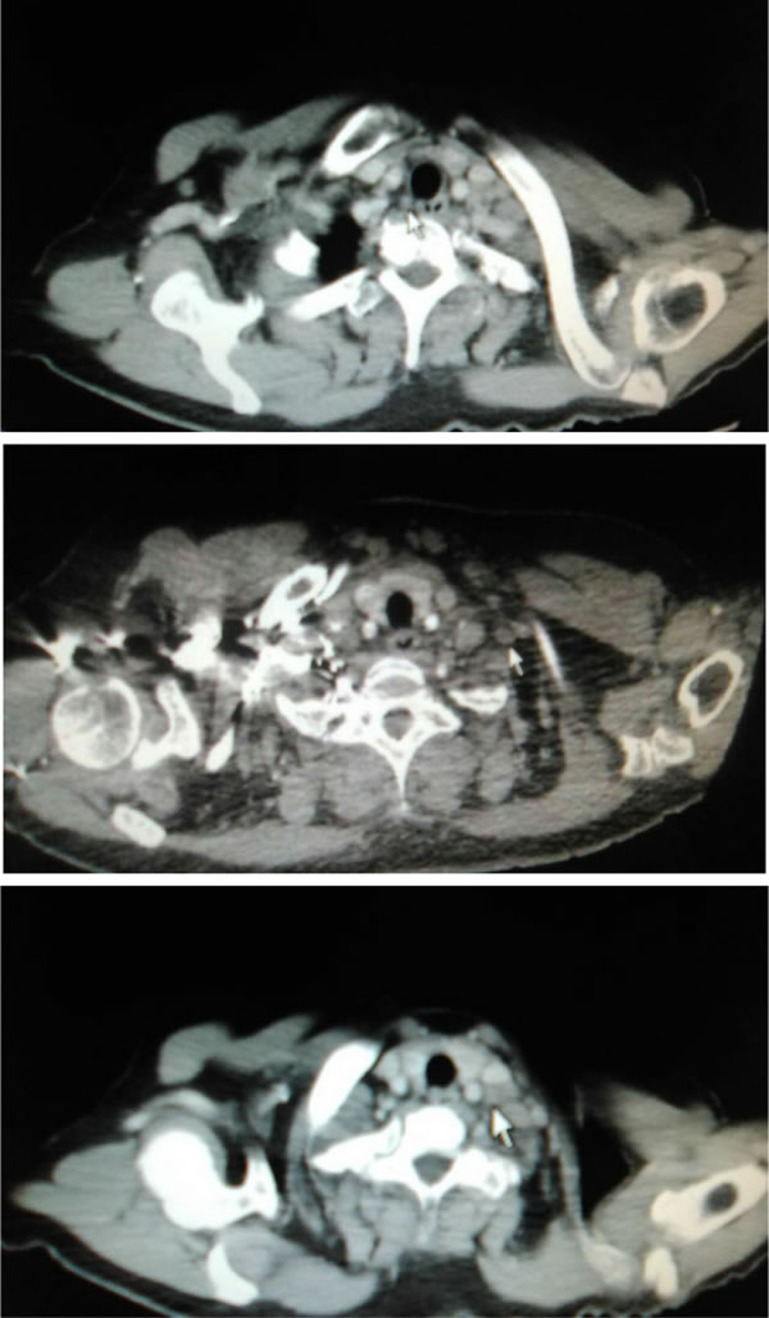
Lymph node metastasis in CTG, MSC and LSC zones, respectively (pointed out by the white arrow)

### Statistical methods

The zone-to-zone metastasis of lymph node was analyzed by means of x2 test. Spearman correlation analysis and Fisher's exact test were used for intergroup comparison. All statistical analyses were performed using SPSS 19.0 statistical software (SPSS Inc, Chicago, IL, USA). P-values<0.05 were considered statistically significant.
